# Biological Anchorage and Canine Orthodontic Movement Rate with a New Technique for Micro-Osteoperforations

**DOI:** 10.1155/2022/5469453

**Published:** 2022-02-03

**Authors:** Andrés Arredondo, Daniela Pérez, Oscar Zapata-Noreña, Claudia Ramírez, Álvaro Carvajal-Flórez, Elsa Arango, Diana Barbosa-Liz, Jorge Gil, Paula Duque, Juan Gallego, Catalina Castaño, Sonia Patricia Plaza-Ruíz

**Affiliations:** ^1^Faculty of Dentistry, University of Antioquia, Calle 70 #52-21 Medellín, Colombia; ^2^Orthopedic Maxillary and Orthodontic Postgraduate Program, Gionorto Research Group, Faculty of Dentistry, Universidad de Antioquia, Calle 70 #52-21 Medellín, Colombia; ^3^Orthodontic Graduate Program, Faculty of Dentistry, Fundación Universitaria CIEO-UniCIEO, Cra 5, #118-10 Bogotá, Colombia

## Abstract

**Introduction:**

The differential management of anchorage and the acceleration of tooth movement are some of the current greatest challenges for orthodontists. Diverse techniques and devices to reinforce anchorage and increase the rate of tooth movement have been proposed. Whether micro-osteoperforations (MOPs) can be used for both purposes is currently investigated.

**Objectives:**

To propose and describe a new technique for biological anchorage, which involves six MOPs performed every four weeks, and to present its results in a clinical case of upper premolar extraction. *Intervention*. In a dental class II patient who met the selection criteria, three MOPs both on the buccal and palatine sides on the intervention side were performed on the extraction area following the protocol described. No MOPs were performed on the control side. The allocation of the intervention was randomised. The MOPs were performed three times at an interval of four weeks. A 0.019 × 0.025-inch stainless steel wire was activated with calibrated NiTi springs. The three-dimensional movement of the first molars and upper canines was evaluated. In addition, the comfort, periodontal status, and canine root resorption of the patient were evaluated.

**Results:**

Clinical and radiographic results suggest that the MOPs had a positive effect in reducing the loss of biological anchorage of the posterior sector and in the rate of canine tooth movement, without damaging changes in the soft and hard tissues.

**Conclusion:**

The proposed protocol involving six MOPs every four weeks improved the behaviour of biological anchorage and increased distalization on the intervention side in this clinical case.

## 1. Introduction

Different methods have been proposed to accelerate the rate of orthodontic tooth movement (OTM) to shorten total orthodontic treatment time. Micro-osteoperforation (MOP) is a surgically assisted method for OTM acceleration. MOPs involve the introduction of microtrauma within the bone, which increases osteoclast activity and the rate of bone remodelling [1] and decreases bone density [2].

Anchorage is defined as resistance to unwanted tooth movement [3]. In orthodontic extraction cases, anchorage control is very important. As osseous tissue must be resorbed for a tooth to move, bone density contributes to greater or lesser “anchorage value” [4, 5]. Therefore, if bone density is reduced and osteoclast activity is increased with MOPs distal to the canine to be retracted to an extraction space, the canine will move rapidly and will need less anchorage reinforcement at the molar area, with less molar mesialization.

Different randomised clinical trials (RCTs) have evaluated the rate of orthodontic canine movement [6–13] and anchorage loss [7, 11, 12] with MOPs and have found conflicting results. Some studies have found that MOPs accelerate the rate of canine retraction [6, 8, 10, 11, 13], whereas others have not found significant differences with control groups [7, 9, 12]. Some authors [14] performed a systematic review of the literature on the effect of MOPs on the rate of orthodontic tooth movement, where anchorage loss is evaluated as a secondary outcome. They found no effect with single-application MOPs over a short observation period and no significant differences in anchorage loss between MOP and control groups in three studies [7] [11, 12]. However, all three studies [7, 11, 12] reinforced anchorage with temporary anchorage devices (TADs) placed mesial to the first molars. These studies had high heterogeneity due to different observation times, outcome measures, and intervention procedure intervals.

In the different studies [6–13, 15], MOPs have been performed to introduce small perforations along the distal and buccal bones of the canine, with variations in the number of perforations (two or three), follow-up periods, and instruments used (propel or orthodontic mini-implants). Regarding frequency, MOPs have been performed only once in some studies [6–8, 10, 12, 15] and every 28 days to 30 days in others [9, 11, 13]. As several authors have suggested, by causing greater injury to bone tissue, it produces less dense and less mature bone that may cause greater acceleration of the OTM; hence, performing MOPs both in the buccal and palatal areas would produce important effects both in distalization of the canine and in the control of the anchorage of the first molar [16]. A study [17] where one of the experimental groups received three buccal and three palatal MOPs showed that an increase in the number of MOPs (from three to six) resulted in a significant acceleration of canine retraction. Given that MOP is a new technique and has not been thoroughly explained, this article is aimed at proposing and describing a new technique for biological anchorage, which involves six MOPs performed every four weeks, and at presenting its results in a clinical case of upper premolar extraction.

## 2. New MOP Technique

1. Perform MOPs using either of the following instruments: (a) mini-implants with a diameter of 1.6 mm to 1.8 mm and a length of 6 mm to 8 mm; (b) Propel® (Propel Orthodontics, Milpitas, CA, USA), a disposable MOP device designed for this purpose that has a stainless steel tip like a mini-implant with a diameter of 1.5 mm
2. Evaluate the area where MOPs will be performed. This evaluation must be both clinical and radiographic to ensure that a root will not be injured
3. Request the patient to rinse his mouth with chlorhexidine for 60 seconds
4. Anaesthetic technique: apply topical anaesthesia to the treatment area and then inject local anaesthetics (2% lidocaine with 1 : 100000 epinephrine)
5. Mark the MOP placement points by bleeding points using a calibrated periodontal probe, with three vertically oriented perforations in a vertical row from the cervical to apical area of the gingiva from the buccal and palatal or lingual side
6. Insert the instrument by rotating the tip of the instrument into the alveolar bone up to a depth of 4 mm and a width of 1.6 mm to 1.8 mm. The first perforation is made buccally from the cervical to the apical part of the edentulous ridge with a gingival direction
7. Remove the instrument
8. Repeat the insertion of the instrument with a distance of 1.5 mm between each MOP until three MOPs each are performed on the buccal side and the palatal side. Repeat the MOP protocol every 28 days
9. Instruct the patients to avoid anti-inflammatory nonsteroidal drugs and only take 250 mg to 500 mg of acetaminophen in case of pain

Given that the use of different techniques in MOP is a main obstacle to comparing different studies, this article is aimed at describing a new technique for biological anchorage with MOPs, which uses six MOPs every four weeks, and at presenting its results in a clinical case of upper premolar extraction.

## 3. Case Report

### 3.1. Diagnostic

This clinical case presented a 14-year-old Colombian mestizo male patient, class II malocclusion with permanent dentition, class II occlusal relationships, 6 mm overjet, and mild upper and lower crowding in good general health and without any systemic or congenital disease. The patient, who after having received extraction of the upper first premolars needed retraction of the upper incisors and canines, was invited to receive the new protocol for biological anchorage with MOPs. The patient and parents accepted to participate, and both he and his guardian were informed about the risks and implications thereof and subsequently gave their informed consent.

### 3.2. Treatment Progress

Fixed orthodontic appliances with an MBT slot 0.022 × 0.028-inch prescription were bonded. The sequence of arches used for alignment and levelling was the conventional one suggested by the MBT philosophy (NiTi 0.014-inch wire, 0.016-inch wire, 0.017 × 0.025-inch wire, and 0.019 × 0.025-inch wire). At the end of the first orthodontic phase, the patient was referred for extraction of upper first premolars, and we proceeded to begin the space closure phase.

At the start of the study, the patient was in the MBT technique working phase, with full alignment and levelling and 0.019 × 0.025-inch steel arches, with a postextraction period of the upper first premolars greater than 3 months and had 8.9 mm of space between the upper right canine and the upper right first premolar 6.9 mm between the upper left canine and the upper left first premolar. In addition, he was periodontally healthy.

**Figure 1:**
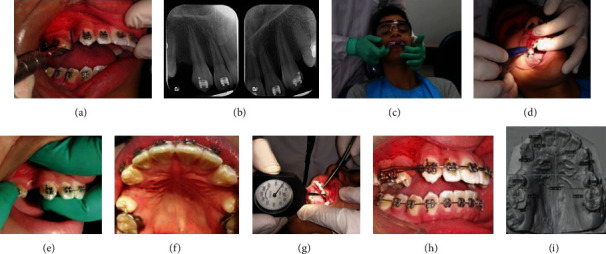
(a) Periodontal exam. (b) Digital periapical radiographs of the canines. (c) Impression taking. (d) Performance of MOPs with Propel®. (e) Buccal MOPs. (f) Palatine MOPs. (g) Measurement of 100 g of force with an ATG-500-1 ALIYIQI® dynamometer. (h) Intervention completed. (i) Digitized upper model.

As part of the preparation for the study, the upper incisors were consolidated with continuous metallic ligature to avoid the opening of diastemas. Subsequently, through the website http://www.randomization.com/, we determined that the intervention and control sides would be the right and left hemimaxillary, respectively. Next, at T0, a periodontal assessment was performed (Figure 1(a)). Subsequently, a digital periapical radiograph was taken from each upper canine using the parallel technique, with a standard KV of 70, an exposure time of 0.25, and a mA of 0.88 (Figure 1(b)). Then, an impression of the upper arch was taken with Orthoprint (Zhermack®) alginate, and the plaster model was immediately fabricated with type III plaster (Whip-mix®) (Figure 1(c)) to obtain a dental cast.

Then, the patient was asked to rinse with 0.02% chlorhexidine for 1 minute, and infiltrative anaesthesia with 2% lidocaine with 1 : 80000 epinephrine was applied. The MOPs were performed on the intervention side using a Propel® Orthodontics device, following the previously described protocol (Figures 1(d)–1(f)). Closed NiTi springs were fitted on both the control and intervention sides, active with 100 grams of force measured with an ATG-500-1 ALIYIQI® dynamometer (Figure 1(g)). Finally, the 0.019 × 0.025-inch stainless steel arch was placed without consolidating the posterior sectors (Figure 1(h)). The patient was then given a 500 mg acetaminophen tablet and a pain and discomfort questionnaire and a visual analogue scale to record pain and discomfort 24 hours after the intervention.

MOPs were performed every four weeks (T1 and T2) during the retraction of individual canines on the intervention side. Finally, in T3, a new periodontal evaluation was performed, a periapical radiograph was taken of each upper canine, and a last alginate impression was made to prepare dental cast, which were digitized for each time point.

**Figure 2:**
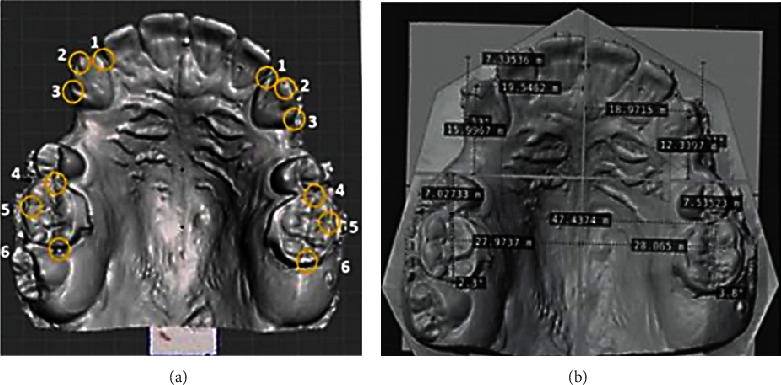
(a) Reference points: (1) most mesial point of the canines; (2) centre of cusp of canines; (3) most distal point of the canines; (4) most mesial point of the first molars; (5) intermediate point between the mesiobuccal and distobuccal of the first molar; (6) most distal point of the first molar. (b) Linear and angular measurements taken from the cross-shaped tool.

The dental cast in the four time points was scanned using the Carestream CS3500® intraoral scanner, generating four STL files. These files were measured and analysed with the Blender® 2.83.3 program. With the median raphe and the entire palatal rugae as a reference, a cross-shaped measuring tool was constructed with a sagittal line that ran through the median raphe and a transverse line perpendicular to it and that passes through the same point in the third palatal rugae in all dental cast. The third rugae have been shown to remain constant with different modalities of orthodontic treatment and can therefore be used as a reliable reference point for measurements in cast models [18–20]. The mesh segment to form the said tool was taken from the T0 cast model, which contained the set of palatal rugae and was joined to the cross tool by using the “Boolean union” action of the Blender program, allowing the superimposition of the tool on each of the other cast models. The mesiodistal, palatal vestibule, and angular movements of the first molars and canines were measured using six dental reference points (Figures 2(a) and 2(b)).

## 4. Results

### 4.1. Dental Movement

The description and comparison of the mean of the absolute distances measured on the intervention side versus the control side showed numerical differences in movement. The intervention side and the control side behaved differently, which may suggest a clinically significant difference between both sides.

**Figure 3:**
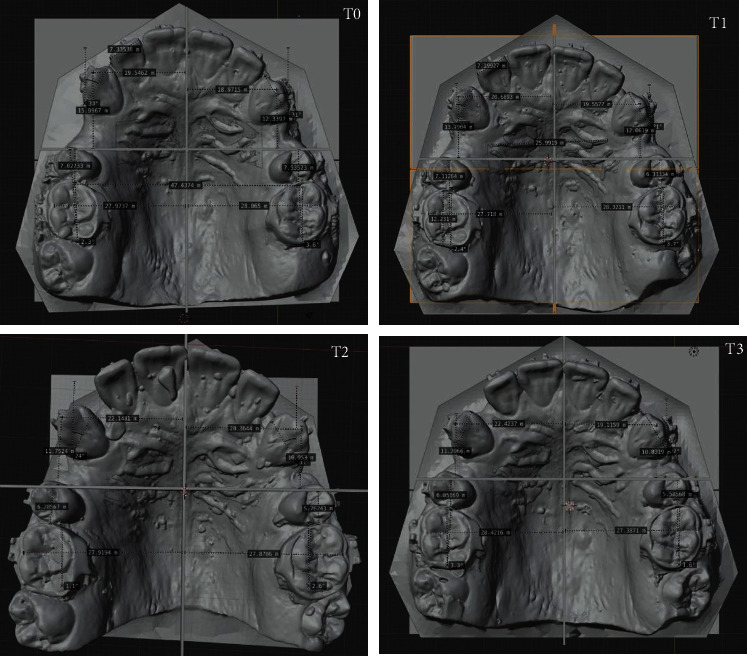
Digitized dental cast from T0 to T4.

Table 1 shows the results of the dental movement measurement in a comparative way at the different time points. An evaluation of the loss of anchorage of the first molars revealed greater loss of anchorage on the control side (left first molar), with a difference of 0.7 mm and with less mesialization of the molar on the intervention side (right first molar). Regarding the sagittal movement of the canines, the intervention side (right) moved 3.21 mm more distally than the control side. Regarding transverse movement, a difference of 1.11 mm was observed between upper first molars, the buccal inclination of upper left first molar (ULFM) being greater (control side). When this same movement was compared for the canines, a difference of 2.67 mm was evident, with a palatine movement of upper right canine (URC, intervention side) and buccal movement of upper left canine (ULC, control side). Finally, an evaluation of the rotational movement of the molars and canines revealed a 1.3° greater mesiobuccal rotation in ULFM and no difference between URC and ULC as both rotated 9° in the mesiobuccal direction. The digital dental model from T0 to T3 is shown in Figure 3.

### 4.2. Periodontal Status

During the observation time, minimal or no changes were found in the periodontal status of the four teeth evaluated both on the control and intervention sides. Only fluctuations were found between a reduced healthy periodontium and gingivitis with reduced periodontium without periodontitis and vice versa, as shown in Table 2. The periodontal evaluation in T3 could not be performed due to the worldwide contingency caused by COVID-19 [21, 22].

### 4.3. Root Status

**Figure 4:**
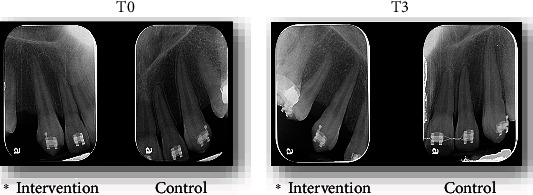
Root length and width measurements of 13 (intervention side) and 23 (control side) in T0 and T3.

Slight root shortening and narrowing were observed in the apical area, as shown in Figure 4. However, these values are comparable, with no clinically significant differences between the intervention side and the control side.

### 4.4. Pain and Discomfort

The patient reported a discomfort score of 4/10 four hours after the intervention in T0, for which he consumed 500 mg acetaminophen once that day. This score decreased in T1, T2, and T3 with values less than 2/10. Regarding the interference of pain in the activities of daily living, the patient reported nonsignificant minimum values.

### 4.5. Surgical Complications

The performance of the MOPs during the intervention was well tolerated by the patient, and no surgical or postoperative complications associated with them were found.

## 5. Discussion

Some studies have been carried out to evaluate the effect of MOPs, their quantity, and their frequency on the acceleration of tooth movement [14, 23]. This minimally invasive surgical technique has been sought to increase the rate of orthodontic tooth movement to reduce treatment time, which is an increasingly evident interest on the part of patients and clinicians, given that a prolonged duration of treatment may be associated with undesired effects, such as periodontal disease, caries, and root resorption; furthermore, it can affect patients' commitment to treatment [24–26].

The present case report is aimed at evaluating the effect of MOPs on the biological anchorage of the upper first molar, which was determined by dental anatomy and periodontal conditions. Current evidence on biological anchorage is limited due to other studies evaluating the effect of MOPs on anchorage by using reinforced anchorage with TADs [7, 12, 26]. An evaluation of the numerical values on the mesial movement of the upper first molars of the patient revealed numerical differences; biological anchorage was lower on the control side (upper left first molar), with a mesial movement of 0.7 mm greater than the intervention side (upper right first molar). This result may indicate that the MOPs, by decreasing bone density [1, 23], allow canine retraction to be performed with less loss of biological anchorage.

Furthermore, the literature shows controversy in the effect of MOPs on the rate of tooth movement during upper canine retraction. Some investigations that performed only three MOPs at the beginning of the RCTs found no differences in the rate of tooth movement between intervention and control sides [7, 12, 27]. Furthermore, studies that increased both the quantity and frequency of MOPs found differences [10, 13, 17, 28, 29]. As the surgical trauma increases, the greater is the stimulation of the regional acceleratory phenomenon, which translates into an increase in inflammatory markers and osteoclastic activity, leading to an increase in the rate of tooth movement [23, 29, 30]. This finding is consistent with our results. When six MOPs were performed every four weeks for three months, the distal movement of the canines increased on the intervention side (right canine), with a difference of 3.21 mm relative to the control side (left canine), which is clinically significant.

The absolute numerical differences when comparing the two sides indicate that the effects of MOPs on the rate of three-dimensional movement of the first molar and upper canine are clinically significant, with greater movement in the canine towards distal and less movement of the molar towards mesial. However, this case report, which addresses a single patient, does not have sufficient statistical power to show effects that are statistically significant. Therefore, the results should be evaluated with caution.

MOPs are considered a minimally invasive surgical method because they are performed without flap elevation and through the use of handheld devices, such as miniscrews and/or Propel®. This could explain why no significant changes have been evidenced in periodontal parameters, such as gingival inflammation and loss of attachment, when the procedure is accompanied by good oral hygiene by the patient. In this case, the observed inflammation was more associated with the presence of local factors (orthodontic bands on first molars) than with MOPs, which is comparable with studies that measured the same variables [12, 27]. This could explain the slight discomfort that this intervention generates in patients. The biggest difference that this new protocol of MOPs introduced is the performance of six MOPs every four weeks in a period of three months, which could lead to relevant pain and discomfort. However, the patient's response showed that as he became more familiar with the intervention, his negative predisposition towards it, as well as his discomfort and pain, diminished to practically negligible importance.

In this case report, slight shortening and thinning of the roots were observed in both canines, although the results did not show clinically significant differences. These observations are similar to those found in other studies that evaluated this parameter with periapical and cone beam tomography [27, 29, 31, 32].

## 6. Conclusions

- The proposed protocol involving six MOPs performed every four weeks showed better behaviour of biological anchorage on the intervention side than in the control side in this clinical case
- A clinically significant increase in molar anchorage and in canine distalization was observed on the side of the MOPs. Similar amounts of root resorption were observed on both sides, although they were not highly significant
- The quantity and frequency of MOPs were shown to have no adverse effects on periodontal health or daily life of patients. The perception of pain was minimal and was observed only during the first hours after the procedure
- A clinical trial of the protocol is required to deny or confirm these findings with sufficient statistical power

## Figures and Tables

**Table 1:** Changes in linear and angular movement of first molars and canines at different time points.

	T0-T1	T1-T2	T2-T3	T0-T3	Mean	SD	T0-T3 Dif
MD-URFM (mm)^∗^	0.05	0.94	0.09	1.09	0.36	0.50	0.7
MD-ULFM (mm)	1.33	0.56	0.18	2.06	0.68	0.58	
MDURC (mm)^∗^	2.48	2	0.45	4.93	1.64	1.05	3.21
MDULC (mm)	0.51	1.09	0.12	1.72	0.57	0.48	
BPURFM (mm)^∗^	0.81	-0.2	-0.5	0.12	0.03	0.68	1.11
BPULFM (mm)	0.31	0.44	0.48	1.23	0.40	0.09	
BP-URC (mm)^∗^	-0.73	-1.7	0	-2.43	-0.81	0.85	2.6
BP-ULC (mm)	-0.19	-0.79	1.23	0.24	0.08	1.03	
ANG-URFM (grades)^∗^	0.2	1.3	-0.3	1.2	0.4	0.81	1.3
ANG-ULFM (grades)	0.1	1.1	1	2.2	0.73	0.55	
ANG-URC (grades)^∗^	-8	4.1	-4.2	-9	-3	4.17	0
ANG-ULC (grades)	0	0	-9	-9	-3	5.19	

^∗^Tooth on the intervention side; MD: mesiodistal movement; BP: buccopalatal movement; ANG: rotational movement; (-): mesiobuccal direction; (+): distobuccal direction; URC: upper right canine; ULC: upper left canine; URFM: upper right first molar; ULRM: upper left first molar.

**Table 2:** Periodontal diagnosis at study times.

Tooth	T0	T1	T2	T3
13^∗^	PSR	PSR	PSR	NA
23	GPR	PSR	GPR	NA
16^∗^	PSR	GPR	GPR	NA
26	GPR	GPR	PSR	NA

^∗^Tooth on the intervention side. PSR: reduced healthy periodontium; GPR: gingivitis in reduced periodontium; NA: not applicable.

## Data Availability

The data used to support the findings of this study are included within the article.
